# Supplementary material for: How Do Football Playing Positions Differ in Body Composition? A First Insight into White Italian Serie A and Serie B Players
Source: J Funct Morphol Kinesiol. 2023 Jun 15;8(2):80. doi: 10.3390/jfmk8020080 (PMC10299074; doi:10.3390/jfmk8020080)
Supplement: Supplementary file 1 [file jfmk-08-00080-s001.zip › jfmk-2431896-supplementary.pdf]

Supplementary material

S1. Post-hoc analysis

Table S1. Height differences. Only statistically significant differences are reported.

group1	group2	difference	p-value	CI lower	CI upper
CF	ES	-8.40	0.001	-11.08	-5.73
CF	MID	-7.87	0.001	-10.69	-5.05
CF	WM	-4.25	0.001	-6.78	-1.77
CF	FB	-6.13	0.001	-8.61	-3.66
CF	SS	-10.25	0.001	-13.12	-7.31
CF	AM	-5.94	0.001	-8.78	-3.10
ES	WM	4.16	0.001	1.60	6.71
ES	CB	8.62	0.001	6.18	11.07
ES	GK	9.81	0.001	7.17	12.46
MID	WM	3.62	0.001	0.92	6.32
MID	CB	8.09	0.001	5.49	10.68
MID	GK	9.28	0.001	6.49	12.06
WM	CB	4.46	0.001	2.18	6.74
WM	GK	5.66	0.001	3.16	8.15
WM	SS	-6.01	0.001	-8.84	-3.18
CB	FB	-6.35	0.001	-8.57	-4.13
CB	SS	-10.47	0.001	-13.20	-7.74
CB	AM	-6.16	0.001	-8.78	-3.54
CB	WM	-4.12	0.001	-6.91	-1.34
GK	SS	-11.66	0.001	-14.57	-8.75
GK	AM	-7.35	0.001	-10.16	-4.55
SS	AM	4.31	0.001	1.20	7.42

GK = goalkeeper, CB = central back, FB = fullback, MID = midfielder, WM = wide midfielder, AM = attacking midfielder, SS = second striker, ES = external strikers, CF = striker.

Table S2. Weight differences. Only statistically significant differences are reported.

group1	group2	difference	p-value	CI lower	CI upper
CF	ES	-10.91	0.001	-13.86	-7.96
CF	MID	-7.78	0.001	-10.88	-4.67
CF	WM	-6.20	0.001	-8.98	-3.41
CF	FB	-7.25	0.001	-9.98	-4.53
CF	SS	-8.82	0.001	-12.06	-5.58
CF	AM	-6.75	0.001	-9.88	-3.63
ES	MID	3.13	0.050	0.00	6.26
ES	WM	4.71	0.001	1.90	7.53
ES	CB	10.21	0.001	7.52	12.90
ES	FB	3.66	0.001	0.90	6.41
ES	GK	12.06	0.001	9.15	14.97
ES	AM	4.15	0.002	1.00	7.30
MID	CB	7.08	0.001	4.22	9.94
MID	GK	8.93	0.001	5.86	11.99
WM	CB	5.50	0.001	2.99	8.01
WM	GK	7.35	0.001	4.60	10.09
CB	FB	-6.56	0.001	-9.00	-4.11
CB	SS	-8.12	0.001	-11.13	-5.12
CB	AM	-6.06	0.001	-8.94	-3.18
FB	GK	8.40	0.001	5.72	11.09
GK	SS	-9.97	0.001	-13.17	-6.76
GK	AM	-7.91	0.001	-10.99	-4.82

GK = goalkeeper, CB = central back, FB = fullback, MID = midfielder, WM = wide midfielder, AM = attacking midfielder, SS = second striker, ES = external strikers, CF = striker.

Table S3. PhA differences. Only statistically significant differences are reported.

group1	group2	difference	p-value	CI lower	CI upper
AM	MID	0.47	0.01	0.06	0.89
ES	GK	-0.37	0.05	-0.74	0.00
FB	GK	-0.35	0.04	-0.69	-0.01
GK	MID	0.48	0.00	0.09	0.86
GK	SS	0.43	0.03	0.02	0.83

goalkeeper = GK, central back = CB, fullback = FB, midfielder = MID, wide midfielder = WM, attacking midfielder = AM, second straiker = SS, external strikers = ES, and striker - CF

Table S4. TBW differences. Only statistically significant differences are reported.

group1	group2	difference	p-value	CI lower	CI upper
AM	CB	3.50	0.00	1.85	5.14
AM	ES	-2.50	0.00	-4.30	-0.71
AM	GK	4.47	0.00	2.71	6.23
AM	CF	3.86	0.00	2.08	5.64
CB	ES	-6.00	0.00	-7.53	-4.47
CB	FB	-3.46	0.00	-4.85	-2.06
CB	MID	-3.95	0.00	-5.58	-2.32
CB	SS	-4.73	0.00	-6.44	-3.02
CB	WM	-3.09	0.00	-4.52	-1.66
ES	FB	2.54	0.00	0.98	4.11
ES	GK	6.97	0.00	5.32	8.63
ES	MID	2.05	0.01	0.27	3.83
ES	CF	6.36	0.00	4.68	8.04
ES	WM	2.91	0.00	1.31	4.52
FB	GK	4.43	0.00	2.90	5.96
FB	CF	3.82	0.00	2.26	5.37
GK	MID	-4.92	0.00	-6.67	-3.18
GK	SS	-5.70	0.00	-7.53	-3.88
GK	WM	-4.06	0.00	-5.62	-2.50
MID	CF	4.31	0.00	2.54	6.08
SS	CF	5.09	0.00	3.24	6.93
CF	WM	-3.45	0.00	-5.03	-1.86

goalkeeper = GK, central back = CB, fullback = FB, midfielder = MID, wide midfielder = WM, attacking midfielder = AM, second straiker = SS, external strikers = ES, and striker - CF

Table S5. ECW differences. Only statistically significant differences are reported.

group1	group2	difference	p-value	CI lower	CI upper
AM	CB	1.29	0.00	0.64	1.94
AM	ES	-1.14	0.00	-1.85	-0.43
AM	GK	1.71	0.00	1.02	2.41
AM	CF	1.37	0.00	0.67	2.08
CB	ES	-2.43	0.00	-3.04	-1.82
CB	FB	-1.37	0.00	-1.92	-0.82
CB	MID	-1.63	0.00	-2.27	-0.98
CB	SS	-1.94	0.00	-2.62	-1.26
CB	WM	-1.18	0.00	-1.75	-0.62
ES	FB	1.06	0.00	0.44	1.68
ES	GK	2.85	0.00	2.20	3.51
ES	MID	0.80	0.01	0.09	1.51
ES	CF	2.51	0.00	1.85	3.18
ES	WM	1.25	0.00	0.61	1.88
FB	GK	1.79	0.00	1.19	2.40
FB	CF	1.45	0.00	0.84	2.07
GK	MID	-2.05	0.00	-2.74	-1.36
GK	SS	-2.36	0.00	-3.08	-1.64
GK	WM	-1.61	0.00	-2.23	-0.99
MID	CF	1.71	0.00	1.01	2.41
SS	CF	2.02	0.00	1.29	2.75
SS	WM	0.75	0.03	0.05	1.46
CF	WM	-1.27	0.00	-1.90	-0.64

goalkeeper = GK, central back = CB, fullback = FB, midfielder = MID, wide midfielder = WM, attacking midfielder = AM, second straiker = SS, external strikers = ES, and striker - CF

Table S6. ICW differences. Only statistically significant differences are reported.

group1	group2	difference	p-value	CI lower	CI upper
AM	CB	2.21	0.00	1.19	3.22
AM	ES	-1.37	0.00	-2.48	-0.25
AM	GK	2.76	0.00	1.67	3.85
AM	CF	2.48	0.00	1.38	3.59
CB	ES	-3.57	0.00	-4.52	-2.62
CB	FB	-2.09	0.00	-2.95	-1.22
CB	MID	-2.32	0.00	-3.33	-1.31
CB	SS	-2.79	0.00	-3.86	-1.73
CB	WM	-1.90	0.00	-2.79	-1.02
ES	FB	1.49	0.00	0.51	2.46
ES	GK	4.12	0.00	3.09	5.15
ES	MID	1.25	0.01	0.14	2.35
ES	CF	3.85	0.00	2.81	4.89
ES	WM	1.67	0.00	0.67	2.66
FB	GK	2.64	0.00	1.69	3.58
FB	CF	2.36	0.00	1.40	3.32
GK	MID	-2.87	0.00	-3.96	-1.79
GK	SS	-3.34	0.00	-4.48	-2.21
GK	WM	-2.45	0.00	-3.42	-1.48
MID	CF	2.60	0.00	1.50	3.70
SS	CF	3.07	0.00	1.92	4.21
CF	WM	-2.18	0.00	-3.16	-1.20

goalkeeper = GK, central back = CB, fullback = FB, midfielder = MID, wide midfielder = WM, attacking midfielder = AM, second striker = SS, external strikers = ES, and striker - CF

Table S7. FFM differences. Only statistically significant differences are reported.

group1	group2	difference	p-value	CI lower	CI upper
AM	CB	5.13	0.00	2.70	7.57
AM	ES	-3.70	0.00	-6.36	-1.04
AM	GK	6.54	0.00	3.93	9.15
AM	CF	5.65	0.00	3.01	8.29
CB	ES	-8.83	0.00	-11.10	-6.56
CB	FB	-5.00	0.00	-7.07	-2.94
CB	MID	-5.77	0.00	-8.19	-3.36
CB	SS	-6.95	0.00	-9.49	-4.41
CB	WM	-4.51	0.00	-6.63	-2.39
ES	FB	3.83	0.00	1.50	6.15
ES	GK	10.24	0.00	7.78	12.70
ES	MID	3.06	0.01	0.42	5.70
ES	CF	9.35	0.00	6.86	11.84
ES	WM	4.32	0.00	1.94	6.70
FB	GK	6.41	0.00	4.15	8.68
FB	CF	5.52	0.00	3.22	7.83
GK	MID	-7.18	0.00	-9.77	-4.59
GK	SS	-8.36	0.00	-11.07	-5.65
GK	WM	-5.92	0.00	-8.24	-3.60
MID	CF	6.29	0.00	3.67	8.91
SS	CF	7.47	0.00	4.73	10.21
CF	WM	-5.03	0.00	-7.39	-2.68

goalkeeper = GK, central back = CB, fullback = FB, midfielder = MID, wide midfielder = WM, attacking midfielder = AM, second straiker = SS, external strikers = ES, and striker - CF

Table S8. FM differences. Only statistically significant differences are reported.

group1	group2	difference	p-value	CI lower	CI upper
AM	GK	1.37	0.04	0.03	2.70
CB	ES	-1.38	0.01	-2.54	-0.22
CB	FB	-1.55	0.00	-2.61	-0.50
CB	MID	-1.31	0.03	-2.54	-0.08
ES	GK	1.82	0.00	0.56	3.08
ES	CF	1.56	0.00	0.28	2.83
FB	GK	1.99	0.00	0.83	3.15
FB	CF	1.73	0.00	0.55	2.90
GK	MID	-1.75	0.00	-3.07	-0.43
GK	SS	-1.61	0.01	-2.99	-0.23
GK	WM	-1.43	0.01	-2.61	-0.24
MID	CF	1.49	0.02	0.15	2.83

goalkeeper = GK, central back = CB, fullback = FB, midfielder = MID, wide midfielder = WM, attacking midfielder = AM, second striker = SS, external strikers = ES, and striker - CF

Table S9. ALST differences. Only statistically significant differences are reported.

group1	group2	difference	p-value	CI lower	CI upper
AM	CB	0.75	0.00	0.40	1.09
AM	ES	-0.39	0.04	-0.77	-0.01
AM	GK	0.90	0.00	0.53	1.28
AM	CF	0.82	0.00	0.44	1.20
CB	ES	-1.14	0.00	-1.46	-0.81
CB	FB	-0.63	0.00	-0.93	-0.34
CB	MID	-0.74	0.00	-1.08	-0.39
CB	SS	-0.96	0.00	-1.32	-0.59
CB	WM	-0.60	0.00	-0.90	-0.29
ES	FB	0.51	0.00	0.17	0.84
ES	GK	1.29	0.00	0.94	1.65
ES	MID	0.40	0.03	0.02	0.78
ES	CF	1.21	0.00	0.85	1.57
ES	WM	0.54	0.00	0.20	0.88
FB	GK	0.79	0.00	0.46	1.11
FB	CF	0.71	0.00	0.38	1.04
GK	MID	-0.89	0.00	-1.26	-0.52
GK	SS	-1.11	0.00	-1.50	-0.73
GK	WM	-0.75	0.00	-1.09	-0.42
MID	CF	0.81	0.00	0.43	1.19
SS	CF	1.03	0.00	0.64	1.42
CF	WM	-0.67	0.00	-1.01	-0.33

goalkeeper = GK, central back = CB, fullback = FB, midfielder = MID, wide midfielder = WM, attacking midfielder = AM, second straiker = SS, external strikers = ES, and striker - CF

Table S10. LLST differences. Only statistically significant differences are reported.

group1	group2	difference	p-value	CI lower	CI upper
AM	CB	1.6907	0.001	0.873	2.5084
AM	ES	-1.2293	0.001	-2.1233	-0.3352
AM	GK	2.146	0.001	1.2696	3.0223
AM	CF	1.86	0.00	0.97	2.74
CB	ES	-2.92	0.00	-3.68	-2.16
CB	FB	-1.62	0.00	-2.31	-0.92
CB	MID	-1.89	0.00	-2.70	-1.08
CB	SS	-2.29	0.00	-3.15	-1.44
CB	WM	-1.48	0.00	-2.19	-0.77
ES	FB	1.30	0.00	0.52	2.09
ES	GK	3.38	0.00	2.55	4.20
ES	MID	1.03	0.01	0.14	1.92
ES	CF	3.09	0.00	2.25	3.92
ES	WM	1.44	0.00	0.64	2.24
FB	GK	2.07	0.00	1.31	2.83
FB	CF	1.78	0.00	1.01	2.56
GK	MID	-2.34	0.00	-3.21	-1.47
GK	SS	-2.75	0.00	-3.66	-1.84
GK	WM	-1.93	0.00	-2.71	-1.15
MID	CF	2.05	0.00	1.17	2.94
SS	CF	2.46	0.00	1.54	3.38
CF	WM	-1.65	0.00	-2.44	-0.85

goalkeeper = GK, central back = CB, fullback = FB, midfielder = MID, wide midfielder = WM, attacking midfielder = AM, second straiker = SS, external strikers = ES, and striker - CF
